# What We Owe to Experiments on Animals

**Published:** 1904-01-02

**Authors:** Stephen Paget


### WHAT WE OWE TO EXPERIMENTS ON ANIMALS.

By Stephen Paget.

### *II.—PATHOLOGY, BACTERIOLOGY, AND THERAPEUTICS.*

(*Continued from page* 224.)

#### III.—Tubercle.

Villemin, in 1865, discovered that tubercle can be transmitted by inoculation from man to animals. Chauveau, in 1880, laid down the rule that "Everything is tuberculous which can produce tuberculous disease, by inoculation, in animals susceptible of that disease; and nothing is tuberculous which cannot do this." Koch, in 1881, discovered the tubercle-bacillus, the cause of all the different forms of tuberculous disease, both medical and surgical. In 1882 he said of this discovery—

"Henceforth, in our warfare against this fearful scourge of our race, we have to reckon *not with a nameless something, but with a definite parasite*, whose conditions of life are for the most part already known, and can be further studied. . . . *Before all things, we must shut off the sources of the infection*, so far as it is in the power of man to do this."

And in these words is the whole meaning of bacteriology, from Pasteur's work on anthrax down to Bruce's recent and amazing discovery of the cause of the African sleeping-sickness—*Henceforth we have to reckon with a definite parasite. Before all things, we must shut off the sources of the infection, so far as it is possible.*

Eight years later, in 1890, came the failure of "Koch's fluid." The disappointment, all the world over, was terrible; but this hope is left, that some modified form of the "old tuberculin" may yet be found which shall not fail. Now let us see what has been done, since 1881, to *shut off the sources of the infection.*

1. By the microscopic examination of sputa, discharges, etc., bacteriology has enabled everybody to diagnose the disease, whether in the lungs or elsewhere, at an early stage, and with great accuracy. The diagnosis may be made by an expert, a hundred miles away from the patient, and the answer sent by telegraph.

2. By experiments on the infectious nature of the sputa of phthisical patients, bacteriology has had a good influence on the nursing of these cases; and it has helped to enforce rules against spitting in public conveyances, etc.

3. It has brought about the systematic use of the microscope, and of inoculations, to prevent the sale of milk containing the bacilli of tubercle.

4. The "old tuberculin" has come into general use for the detection of tuberculosis in cattle. In a healthy animal, there is no reaction after an injection of tuberculin; but in a tuberculous animal, even though the disease be slight, the injection causes a rise of temperature. Of this simple test Professor McFadyean, Principal of the Royal Veterinary College, says: "Using it on animals in their own premises, I have found that it is practically infallible. I have notes of one particular case where 25 animals in one dairy were tested, and afterwards all were killed. There was only one animal which did not react, and it was the only animal not found to be tuberculous when killed."[1]

(In 1901 Koch stated that human tuberculosis and bovine tuberculosis are not one and the same disease, and that the risk of infection from animals to man is

[1] For other references see Lancet, Aug. 5, 1899; and Gould's Year-book of Medicine and Surgery, 1902.

nothing, or next to nothing ; and he was in favour of some relaxation of the present strict supervision of the sale of meat and milk. These views were met by strong opposition ; and it is best, meanwhile, to be on the safe side. Seeing that many people, especially among the poor, do not boil the day's milk, and seeing that *tabes mesenterica*—tuberculosis of the abdominal glands—which every year kills about 7,000 children in England alone, is, or may be due, in many cases, to the milk of tuberculous cows—we could hardly view with indifference the unhindered sale of milk *plus* tubercle-bacilli.)

The opponents of all experiments on animals must face these facts. The early and positive diagnosis of phthisis has become a part of the day's work, both in hospital practice and in private practice. The detection of phthisis and other forms of tuberculosis in cows is in the hands of the sanitary authorities. The testing of milk is ordered by the county councils and the municipal corporations, and involves about a thousand inoculations annually in England. In some instances it is enough if the microscope be used ; in some it is advisable to inoculate a guinea-pig or a rabbit ; but both methods alike go straight back to those first inoculations which proved that tubercle is transmissible, and that it is due to a specific micro-organism. At the present time, the bacteriology of tubercle has become, in great part, a national affair, a matter of public health : the bacteriologists, like the public analysts, are working under orders : and every inch of their work is the direct outcome of experiments on animals, and involves experiments on animals. Where will the opponents of all these experiments make their attack ? Departments of State, county councils, municipal corporations, medical officers of health, sanitary inspectors—they are all of them actively engaged, as it were with licenses and with certificates to dispense them from giving anæsthetics, in the inoculation of animals, or at any rate in work that goes straight back to the inoculation of animals.

### IV.—Diphtheria.

As with tubercle, so with diphtheria, bacteriology has become (thank Heaven for it) a sort of public servant, or minister of national health. Thus, in 1901, no less than 2,636 inoculations were made for the testing of antitoxins ; and the general consumption of diphtheria-antitoxin can only be met by the incessant " manufacture " of the serum in very large quantities.

The bacillus of diphtheria was discovered by Klebs in 1875, and isolated in pure cultures by Loeffler in 1884. About 1890, Behring and Kitasato succeeded in immunising animals against it ; and in 1893 came news of the first cases treated with antitoxin. About the end of 1894 the antitoxin came into use at the hospitals of the Metropolitan Asylums Board. Before we take the results at those hospitals, let us look further afield.

Where shall we begin ? Paris, Berlin, Munich, Strasburg, Rome, Vienna, Boston, Chicago, or New York ? Shall we reckon in thousands, or in tens of thousands ? Shall we take the experiences of individual men, or the statistics of nations ? Look where we will, ask whom we will, it all comes to the same fact, that we have here the most blessed drug that has been revealed to the world since the discovery of anæsthetics. Let us begin with some early results, taken almost at random, from other countries.

1. In 1894, Roux published the first antitoxin results from the Paris Hospital for Sick Children Before antitoxin, the case mortality from diphtheria in that hospital had been as follows : in 1890, 55·88 per cent. ; in 1891, 52 45 ; in 1892, 47·64 ; in 1893, 48 47. Average mortality per 100 cases during 1890–1893 = 51·71. In 1894, from February to July, 320 children were admitted with diphtheria. In every case the Klebs-Loeffler bacillus was found ; all other cases are excluded. Of the 320 children, 20 were dying on admission, and were not treated with antitoxin. Of the 300 who were treated with it, 78 died ; case-mortality 26 per cent. *During the same period*, at another Paris hospital, where the serum was not used, they had 520 cases, with 316 deaths ; case-mortality 60 per cent.

2. In 1895, at Berlin, during an epidemic of diphtheria, the supply of antitoxin ran short. " In Baginsky's clinic," says the *British Medical Journal*, Oct. 20, 1895, " the interruption of the serum treatment promptly raised the mortality from 15·6 to 48·4 per cent."

3. In 1896, at Montreal, a committee of collective investigation reported on a large number of antitoxin cases, treated not in hospitals but in private practice—no less than 3,384 cases, treated by 613 doctors, in 114 towns in the United States, Columbia, and Canada, *plus* 2,410 cases treated in tenement houses in Chicago and New York. Of these 5,794 cases, 218, *i.e.* 3·7 per cent., were already so near death that the serum—if the phrase may be allowed—never had a chance. Of the 5,576 cases where it did have a chance, 996 were treated on the first day of the disease, of whom 49 died = 4·9 per cent. ; 1,616 on the second day, of whom 120 died = 7·4 per cent. ; 1,508 on the third day, of whom 134 died = 8.8 per cent. ; 758 on the fourth day, of whom 147 died = 20·7 per cent. ; 690 on or after the fifth day, of whom 244 died = 35·3 per cent. ; and in 232 cases the day of the disease was not known. Thus the earlier the serum was given, the more lives it saved.

4. In 1898, the medical report of the French Army stated that, since the introduction of the serum treatment of diphtheria, the mortality among cases of that disease had fallen from 11 per cent. to 6 per cent. (*Brit. Med. Journ.*, Sept. 3, 1898.)

5. In 1898, an analysis was made of the ratio of mortality in 266 German cities of about 15,000 inhabitants. It was found that the ratio of mortality per 100,000 of the living, before antitoxin was used, varied from 130 to 84 from 1886 to 1893, and the ratio from 1894 to 1897 varied from 101 to 35. " It is a significant fact that during 1894, when, although antitoxin was used to a certain extent, it was not in general use, the ratio was 101 ; that when antitoxin was used more extensively, in 1895, the ratio was 53 ; that in 1896 it was 43 ; that in 1897, when antitoxin was very generally used, the rate fell to 35." (*Trans. Massachusetts Med. Soc*, 1898.)

6. In 1898, at the German Surgical Congress, Professor Krönlein, of Zürich, reported his experience of the serum. " In his clinic all the patients were examined bacteriologically, and serum was administered in every case of diphtheria without exception. Of 1,336 cases treated before the serumperiod, 554=39·4 per cent. died; while during the serum-period there were 55 deaths among 437 cases =12 per cent. In cases of tracheotomy, the death-rates before and during the serum-period were 66 and 38·8 per cent. respectively." (*Lancet*, May 7, 1898.)

Here, from diverse parts of the world, are six instances; and it would be easy to give six hundred. Or we might be content with Siegert's colossal tables (1900), which deal with 40,038 cases, in 69 hospitals in Germany, France, Austria, and Switzerland, from 1890 to 1898. But let us come nearer home, and take the statistics of the hospitals of the Metropolitan Asylums Board.[1] Out of 100 diphtheria cases, in the years before antitoxin, how many died? Out of 100 cases, in the years after antitoxin, how many died? Diphtheria cases were first admitted in 1888; that year therefore is reckoned incomplete :—

*Case-mortality per* 100 *cases, in all the Hospitals taken together.*

| | | | | | |
|---|---|---|---|---|---|
| 1888 | (incomplete) | 59 35 | 1895 | (first anti-toxin year) | 22·55 |
| 1889 | ... ... | 40 74 | 1896 | ... ... | 20 80 |
| 1890 | ... ... | 33 55 | 1897 | ... ... | 17·50 |
| 1891 | ... ... | 30·63 | 1898 | ... ... | 15·50 |
| 1892 | ... ... | 29 35 | 1899 | ... ... | 14·05 |
| 1893 | ... ... | 30·42 | 1900 | ... ... | 12 01 |
| 1894 | ... ... | 29 64 | | | |

Case-mortality, of course, is not the same thing as general mortality. The tables of mortality published by the Registrar-General show how heavily diphtheria lies on the land, especially of late years: and the antitoxin can no more *prevent* a bad diphtheria-year than an umbrella can prevent a wet day, or a field-hospital prevent a battle. It *can* be used as a preventive; a dose of the serum all round is a good protection for children exposed to sudden danger of infection in a small and close community, in a family, in a school, or in a village:[1] but this *preventive* use of the antitoxin is at present too small to have any appreciable effect on the Registrar-General's returns.

The lowering of the case-mortality since 1894 cannot be explained away by any theory that the disease suddenly became mild. First, because the general mortality has gone up, not down. Next, because the antitoxin results were the same everywhere, all at once, and all over the world; and the clinical aspect of the disease, under the new treatment, was changed, alike in mild cases and in bad cases. Next, because world-diseases do not behave in this way of their own accord. Next, because the fact that animals can be immunised against diphtheria has been proved past all possibility of doubt.

In 1901, two grievous instances occurred of contamination of a store of the antitoxin. These disasters happened far from this country; but the lesson of them will not soon be forgotten here.

The slight drawbacks that, in some cases, attend the treatment, are not to be considered in the fight against this terrible disease.[2]

A curious American anti-antitoxin journal, called the *Medical Brief*, says that carbolic acid is the real agent in the antitoxin. This silly statement may be left to those people who can believe it. The *Zoophilist*, the "official journal" of the National Anti-vivisection Society, is very fond of this bit of nonsense out of the *Medical Brief*; and says that the acid "acts antiseptically in the blood, and so assists nature in getting rid of the decomposing corpuscles." Let us commend this phrase to the proprietors of Mother Siegel's Syrup.

[1] For a full account of these statistics, with references to reports, etc., see "Experiments on Animals," pages 137–149.

[1] For striking instances of this protective use of the antitoxin, see Brit. Med. Journ., Jan. 16, 1897; Lancet, April 2, 1898, and Jan. 28, 1899; Boston Medical and Surgical Journal, Dec. 1897, and March, 1898; and, especially, Gould's Year-book, 1902.

[2] That the serum is not the cause of anything worse than slight and fugitive troubles, is proved by Woodhead, in his monumental report (1901). See also Dr. Woollacott's admirable monograph on this subject, Lancet, August 26th, 1899.

(*To be continued.*)

